# High expression of ID1 in monocytes is strongly associated with phenotypic and functional MDSC markers in advanced melanoma

**DOI:** 10.1007/s00262-019-02476-9

**Published:** 2020-01-17

**Authors:** Jeroen Melief, Yago Pico de Coaña, Roeltje Maas, Felix-Lennart Fennemann, Maria Wolodarski, Johan Hansson, Rolf Kiessling

**Affiliations:** 1 Department of Oncology-Pathology, Karolinska Institute, Visionsgatan 4, 171 64 Solna, Stockholm, Sweden; 2 Department of Oncology, Ludwig Institute for Cancer Research, University of Lausanne, Lausanne, Switzerland; 3 Department of Tumor Immunology, Institute for Molecular Life Sciences, Radboud University Medical Center, Nijmegen, The Netherlands; 4 Karolinska University Hospital Solna, Stockholm, Sweden

**Keywords:** Myeloid cells, Immunosuppression, Cancer, Melanoma

## Abstract

The efficacy of immunotherapies for malignant melanoma is severely hampered by local and systemic immunosuppression mediated by myeloid-derived suppressor cells (MDSC). Inhibitor of differentiation 1 (ID1) is a transcriptional regulator that was shown to be centrally involved in the induction of immunosuppressive properties in myeloid cells in mice, while it was overexpressed in CD11b^+^ cells in the blood of late-stage melanoma patients. Therefore, we comprehensively assessed ID1 expression in PBMC from stage III and IV melanoma patients, and studied ID1 regulation in models for human monocyte differentiation towards monocyte-derived dendritic cells. A highly significant elevation of ID1 was observed in CD33^+^CD11b^+^CD14^+^HLA-DR^low^ monocytic MDSC in the blood of melanoma patients compared to their HLA-DR^high^ counterparts, while expression of ID1 correlated positively with established MDSC markers S100A8/9 and iNOS. Moreover, expression of ID1 in monocytes significantly decreased in PBMC samples taken after surgical removal of melanoma metastases, compared to those taken before surgery. Finally, maturation of monocyte-derived DC coincided with a significant downregulation of ID1. Together, these data indicate that increased ID1 expression is strongly associated with expression of phenotypic and immunosuppressive markers of monocytic MDSC, while downregulation is associated with a more immunogenic myeloid phenotype. As such, ID1 may be an additional phenotypic marker for monocytic MDSC. Investigation of ID1 as a pharmacodynamic biomarker or its use as a target for modulating MDSC is warranted.

**Electronic supplementary material:**

The online version of this article (10.1007/s00262-019-02476-9) contains supplementary material, which is available to authorized users.

## Introduction

Current immunotherapies for malignant melanoma mostly aim to promote T-cell-mediated immune responses against the tumor, for example via checkpoint blockade, adoptive transfer of T cells, or vaccination strategies. Even though these approaches have improved overall survival rates in melanoma, many immunotherapies still display limited efficacy on their own [1]. Importantly, anti-tumor immune responses are severely hampered by tumor-residing and circulating immature myeloid cell populations, such as myeloid-derived suppressor cells (MDSC) and immature dendritic cells (DC) [2]. The clinical importance of this is underlined by the finding that numbers of MDSC are negatively correlated with survival of melanoma patients and can be used as a predictive marker of therapeutic response to ipilimumab [3–7]. Therefore, novel approaches to modulate immature myeloid cells are strongly warranted, to overcome immunosuppression, and achieve more effective T-cell mediated anti-tumor responses by immunotherapies against melanoma.

MDSC express a variety of surface-bound and secreted factors to suppress T-cell-mediated immunity, such as inducible nitric oxide synthase 2 (iNOS), arginase 1 (ARG1), and programmed death-ligand 1 (PD-L1) [2, 8]. An attractive approach to block MDSC-mediated immunosuppression would be to target the molecular mechanisms that govern MDSC formation. One very good example in this respect is blockade of prostaglandin E2 (PGE2) secretion, which was shown to prevent induction of an MDSC-like phenotype in human monocytes [9]. Interestingly, it was found in melanoma mouse models that tumor cells through TGF-β production can promote MDSC formation by induction of the transcriptional regulator called inhibitor of differentiation 1 (ID1) [10]. Additionally, ID1 mRNA levels in CD11b^+^ myeloid cells from peripheral blood mononuclear cells (PBMC) of melanoma patients were increased in comparison to those from healthy donors [10]. Moreover, increased ID1 expression in tumors has been associated with poor outcome in breast, esophageal, and pancreatic cancers [11–13].

ID1 is a helix–loop–helix (HLH)-shaped transcriptional regulator that dimerizes with other HLH proteins, predominantly E proteins, and thereby inhibits their function as transcription factors [12, 14]. ID1 is associated with regulation of endothelial cell differentiation and angiogenesis, as well as mobilization of endothelial cells, which can aid in tumor survival and metastasis [15, 16]. In mice, overexpression of ID1 in bone-marrow cells caused systemic immunosuppression by downregulation of molecules crucially involved in DC differentiation and led to MDSC expansion [10, 17]. Conversely, ID1 knockdown favors expansion of myeloid cells with a DC phenotype and decreased numbers of MDSC [10]. These data suggest that ID1, at least in mice, regulates immunosuppression by controlling a phenotypic switch from DC to MDSC in myeloid cells. As such, ID1 may serve as novel therapeutic target for skewing myeloid cells towards a less immunosuppressive and more immunogenic phenotype in cancer patients.

Interestingly, ID1 is thought to promote MDSC development through upregulation of S100A8/9, a relatively new MDSC marker [10]. S100A8/9 consists of a heterodimer of the calcium-binding pro-inflammatory proteins S100A8 and S100A9, the latter of which has been suggested to be a novel murine and human MDSC marker by itself [18]. Expression of S100 family members in tumors leads to more aggressive outgrowth and metastasis [19], while their expression in myeloid cells is associated with hampered DC differentiation and enhanced MDSC formation [20, 21]. S100A8/9 is overexpressed in MDSC in different types of cancer and its expression is correlated with tumor load [18, 20, 22–24]. Therefore, a positive correlation between ID1 and S100A9, and S100A8/9 would be expected. However, there is some controversy regarding this relation, as it has also been shown that ID1 downregulates S100A9 in breast cancer and promotes formation of metastasis [25]. Here, we aim to further unravel the relation between ID1 and downstream regulators such as S100A8/9 and S100A9, and investigate whether ID1 may, indeed, be centrally involved in the biology of suppressive myeloid cells.

## Materials and methods

### Patient cohort

A total of 24 advanced stage melanoma patients undergoing surgical removal of resectable metastatic lesions were included. Blood samples of the participants were collected prior to surgery of the melanoma lesions and after a median of 35 days post-surgery (range 14–119 days). The median age of patients at the time of surgery was 63 years (range 44–87 years). An overview of additional patient characteristics is shown in Table 1. Patients did not receive systemic therapy prior to or during the period of sample collection. Samples were analyzed using flow cytometry, in which monocytic MDSC were defined as CD33^+^CD11b^+^CD14^+^HLA-DR^low^ (see Supplementary Fig. 1 for a full gating strategy).

**Table 1:** Overview of patient characteristics

Variable	Number of patients (%)
Sex	
Male	17 (71)
Female	7 (29)
Stage	
IIIB	9 (38)
IIIC	7 (29)
IV	8 (33)
T category*	
TX/T0	6 (25)
T1	3 (13)
T2	5 (21)
T3	5 (21)
T4	5 (21)
BRAF status	
V600	6 (25)
WT	12 (50)
Unknown	6 (25)

*Evaluated according to the TNM classification systems

### Peripheral blood samples

Patient-derived peripheral blood samples were acquired via the Oncology Department of the Karolinska University Hospital. Blood samples from healthy donors were obtained from the University lab at the Karolinska University Hospital. PBMC were extracted from peripheral blood samples via Ficoll density gradient centrifugation (Ficoll-Paque plus, GE Healthcare Life Science). Patient-derived and healthy donor PBMC were cryopreserved in fetal bovine serum (FBS) with 10% DMSO. PBMC were thawed for analyses by flow cytometry or DC maturation at a later time point.

### Monocyte isolation

Isolation of monocytes from fresh or thawed human PBMC was performed using magnetic activated cell sorting (MACS) according to the manufacturer’s instructions (Miltenyi Biotec Cat. No. 130-024-210). Monocytes were isolated using CD14 + microbeads (Miltenyi Biotec Cat. No. 130-050-201) and resuspended in IMDM 10% human AB serum.

### DC maturation

Per well 1 × 10^6^ isolated monocytes where plated in 12-well plates (TPP) in 1 ml IMDM with 10% human AB serum (Karolinska University Hospital). Differentiation of monocytes to immature DC (iDC) was done using a fast protocol, in which iDC formation was established by 48 h of culture in the presence of 100 ng/ml GM-CSF (Peprotech) and 20 ng/ml IL-4 (Peprotech). Mature DC (maDC) were created by incubating iDC for an additional 18 h with one of the three following cocktails. The first was the gold standard [26]: 20 ng/ml tumor necrosis factor-α (TNF-α; Peprotech), 10 ng/ml interleukin-1β (IL-1β; CellGenix), 1000 U/ml interleukin-6 (IL-6; CellGenix), and 10 ng/ml PGE2 (SIGMA). Second, the alpha-type 1 polarizing cocktail [27]: 50 ng/ml TNF-α, 25 ng/ml IL1b, 3000 U/ml IFN-α (R&D Systems), 100 U/ml IFN-γ (Imukin^®^, Boehringer Ingelheim), and 250 ng/ml polyinosinic:polycytidylic acid (poly I:C, Sigma-Aldrich). Finally, the COMBIG CCK Cocktail [28] was used: 10 ng/ml LPS (Sigma-Aldrich), 20 μg/ml Hiltonol (OncoVir), 2.5 ug/ml R848 (VacciGrade™, InvivoGen), and 1000 U/ml IFN-γ. Readout was performed by flow cytometry.

### Antibodies and flow cytometry

Single-cell solutions were stained with fluorescent-activated cell sorting (FACS) antibodies to measure protein expression levels. 0.2 × 10^6^ cells were plated in 96-well plate (V-bottom) and washed with PBS. Cells were blocked with 1 µl of IvIgG (Privigen, Germany) for 5 min at room temperature. Next, antibodies for staining of surface markers and dead cell marker were added in PBS in a total volume of 20 µl and kept at 4 °C for 30 min. The following extracellular antibodies were used; CD33 PE-CF594 (BD Biosciences), CD86 PE-Cy7 (Biolegend), HLA-DR APC-Cy7 (Biolegend), CD80 BV421 (Biolegend), Aqua Dead Cell Marker (Life Technologies), CD14 BV570 (Biolegend), CD11b BV605 (Biolegend), and PD-L1 BV786 (BD Biosciences). Samples were washed with FACS buffer (PBS with 1% FBS) and treated for 40 min at room temperature in the dark with 100 µl FoxP3 Fix/Permbuffer (eBioscience). After washing with Perm-wash buffer, samples were stained with intracellular antibodies in Perm-wash buffer in a volume of 20 µl. Staining took place for 40 min at room temperature in the dark. The following intracellular antibodies were used; S100A8/9 FITC (BMA Biomedicals), S100A9 FITC (Biolegend), ID1 PE (LSBio), iNOS PerCP (Santa Cruz Biotechnology), IDO PE-Cy7 (eBioscience), IRF8 APC (eBioscience). Samples were washed three times with Perm-Wash buffer and diluted in 150 µl FACS buffer prior to read-out on the Novocyte Flow cytometer (ACEA Biosciences, Sweden). Analysis was performed using FlowJo (v.10.0.7, Tree Star Inc.). Median or geometrical mean fluorescence intensity (MFI and geoMFI respectively) was used for measurement of protein expression.

### Statistical analysis

GraphPad Prism software (version 6.0) was used for both statistical and graphical analysis. For data analysis, Wilcoxon matched-pair signed-rank test was used. Correlation calculations were performed using a Spearman test. *p* values < 0.05 were considered significant.

## Results

### ID1 expressing cells in melanoma patients have an immunosuppressive phenotype.

**Fig. 1:**
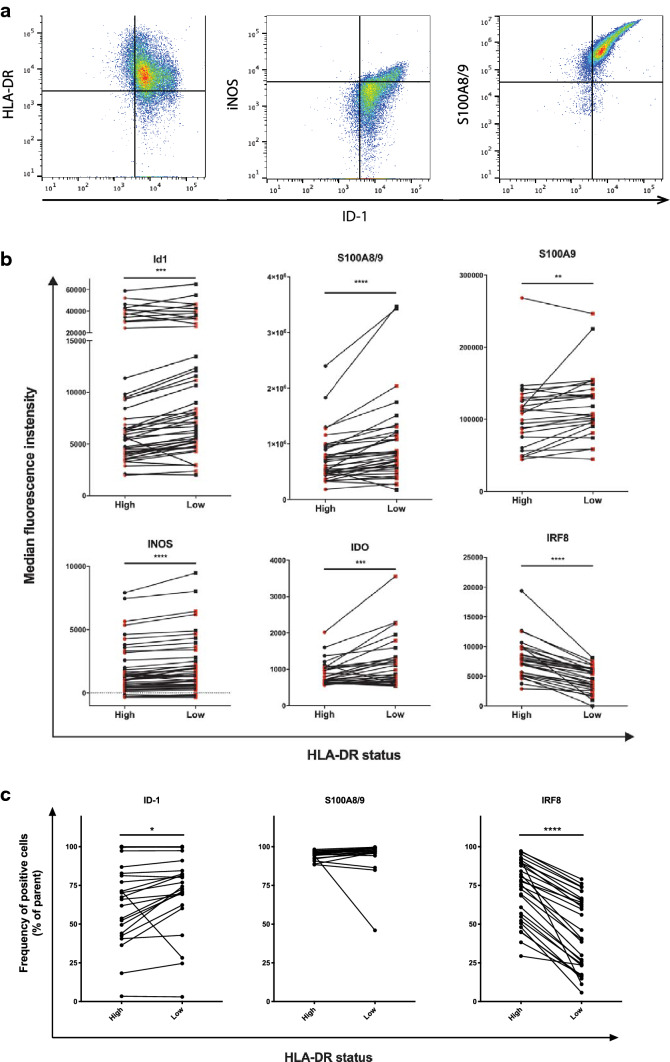
Expression of ID1 on monocytes coincides with known phenotypic characteristics of monocytic MDSC. **a** Flow cytometric analysis of PBMC from melanoma patients. Doublets were excluded and live PBMC were gated (not shown). Representative plots depicting the subpopulation of CD33^+^CD11b^+^CD14^+^ cells, indicating expression of ID1 plotted against markers commonly used for characterization of monocytic MDSC, with gates to indicate cells positive for ID1, HLA-DR, iNOS, and S100A8/9. **b** Flow cytometric analysis of CD33^+^CD11b^+^CD14^+^ cells within melanoma patient PBMC, indicating median fluorescence intensities in HLA-DR^high^ monocytes versus HLA-DR^low^ monocytic MDSC for ID1, S100A8/9, S100A9, iNOS, and IRF8. **c** Frequencies of cells positive for ID1, S100A8/9, and IRF8 with HLA-DR^hi^ and HLA-DR^low^ monocytes. **p < 0.01; ***p < 0.001; ****p < 0.0001

As ID1 has been mostly studied in mouse MDSC, we first set out to study in more detail how the expression of known MDSC markers relates to ID1 expression in human monocytic cells [3, 5, 6, 11, 29, 30]. In addition, we investigated to what extent the expression of these markers is affected by a reduction in the tumor burden after surgical removal of melanoma metastases. Therefore, we studied peripheral blood samples collected from 24 stage III and IV melanoma patients. In these samples, we studied ID1 expression in parallel with more established MDSC markers, to evaluate to what extent ID1 can serve as an accurate marker to distinguish HLA-DR^low^ monocytic MDSC from normal HLA-DR^high^ monocytes in humans. For a full gating strategy, see Supplementary Fig. 1. Low-to-negative expression of HLA-DR on CD33^+^CD11b^+^CD14^+^ monocytes was defined using the lymphocyte population as an internal control, as the bulk of these cells are negative for HLA-DR. A subpopulation of activated T cells may express HLA-DR at a relatively low level, which was also seen in our samples. We started out by studying levels CD33^+^CD11b^+^CD14^+^ cells for expression of ID1 in relation to markers commonly used for characterization of monocytic MDSC: HLA-DR, iNOS, and S100A8/9. Within the population of CD33^+^CD11b^+^CD14^+^ monocytic cells, we found that the highest expression of ID1 was consistently found in HLA-DR^low^ cells. At the same time, cells with higher ID1 expression were also more positive for iNOS and S100A8/9 in the same subpopulation of CD33^+^CD11b^+^CD14^+^ cells (Fig. 1a). Interestingly, HLA-DR^low^ monocytic MDSC displayed a highly significant increase in ID1 expression compared to normal HLA-DR^high^ monocytes, which coincided with strongly increased levels of S100A8/9 and S100A9 (Fig. 1b). Moreover, iNOS and IDO, two mediators of immunosuppression, were both significantly increased in HLA-DR^low^ monocytic MDSC, indicative of an immunosuppressive phenotype (Fig. 1b). Finally, HLA-DR^low^ monocytic MDSC exhibited a strong reduction in IRF8 expression compared to HLA-DR^high^ monocytes (Fig. 1b). In line with these data, we found that HLA-DR^low^ cells contained significantly higher frequencies of ID1-positive cells and significantly lower frequencies of IRF8-positive cells (Fig. 1c). No differences could be found for frequencies of cells positive for S100A8/9, however. This is almost certainly caused by the fact that in the large majority of patient samples virtually all monocytes are S100A8/9 positive, whereas S100A8/9 expression levels vary substantially, as illustrated by the S100A8/9 data shown in Fig. 1b.

None of the analyzed markers correlated significantly with the percentages of MDSC in the total cell population, except for HLA-DR (*r* = − 0.77, *p* = 0.0001). No inverse correlation between ID1 and IRF8 was present in our samples, even though ID1 was reported to induce MDSC differentiation at least partly by downregulating IRF8 in mice [10].

**Fig. 2:**
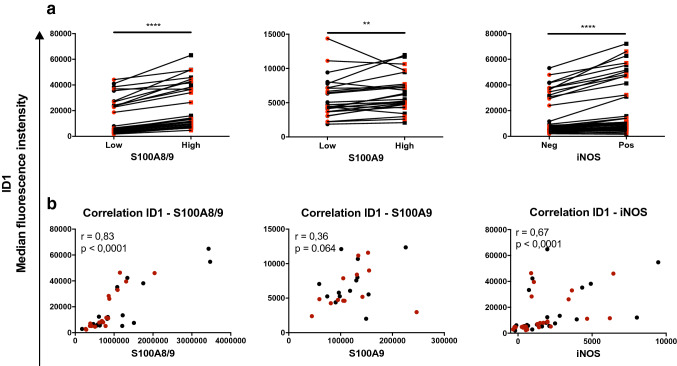
High expression of ID1 on monocytes correlates with expression of known phenotypic characteristics of monocytic MDSC. **a** Flow cytometric analysis of CD33^+^CD11b^+^CD14^+^ cells amongst melanoma patient PBMC. **a** Median fluorescence intensities of ID1 in monocytes comparing various subpopulations of cells within the CD33^+^CD11b^+^CD14^+^ gate: S100A8/9^low^ versus S100A8/9^high^ cells, S100A9^low^ versus S100A9^high^ cells, and iNOS^high^ versus iNOS^low^ cells. **b** Correlations between ID1 and either S100A8/9, S100A9 or iNOS. Depicted are *r* and *p* values of Spearman’s rank correlations. Black and red dots represent samples taken before and after surgery, respectively. **p < 0.01; ***p < 0.001; ****p < 0.0001

To get additional evidence for a relation between ID1 expression and expression of more established MDSC markers, we subdivided monocytic cells in S100A8/9^hi^ and S100A8/9^low^ cells, while the same was done for S100A9 and iNOS. We found that S100A8/9^high^, S100A9^high^, and iNOS^high^ monocytes all expressed significantly higher levels of ID1 compared to, respectively, S100A8/9^low^, S100A9^low^, and iNOS^low^ (Fig. 2a). This was further confirmed by the fact that ID1 expression correlated positively with S100A8/9 (*r* = 0.83, *p* < 0.0001) and iNOS (*r* = 0.67, *p* < 0.0001), but not significantly with S100A9 (*r* = 0.36, *p* = 0.0640), as shown in Fig. 2b.

### Expression of ID1 in monocytes decreases after surgical removal of melanoma metastases

**Fig. 3:**
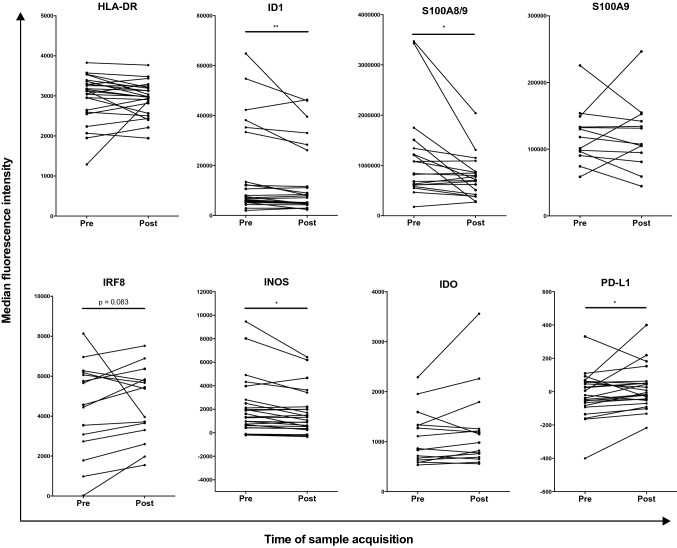
Flow cytometric analysis of PBMC from melanoma patients before and after surgical removal of melanoma metastases. Depicted are median fluorescence intensities of the indicated markers in monocytes, defined as CD33^+^CD11b^+^CD14^+^. *p < 0.05 **p < 0.01

When comparing patient samples before and after surgery, the resection of tumor tissue had no apparent effect on percentages of circulating monocytic MDSC (Supplementary Fig. 2). Interestingly, ID1, S100A8/9, and iNOS expression were significantly decreased after surgery in CD33^+^CD11b^+^CD14 monocytes (Fig. 3). In contrast, an increase in expression after surgery was present for PD-L1 (Fig. 3). With near significance, a trend towards an increase was seen for IRF8 after surgery (Fig. 3). In addition to this, the frequency of cells positive for ID1 and iNOS decreased within the monocytes population after surgery (Supplementary Fig. 3). When analyzing the total frequency of monocytic MDSC positive for the studied markers within all live cells, no changes were observed after surgical removal of melanoma metastases (Supplementary Fig. 4).

### ID1 is downregulated during DC maturation

**Fig. 4:**
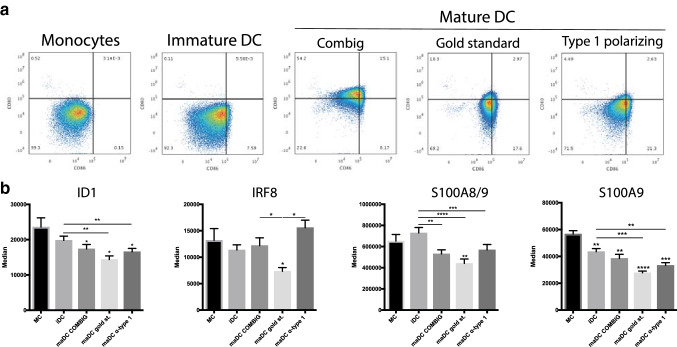
ID1 is downregulated during DC maturation. **a** Flow cytometric analysis of human monocytes and monocyte-derived dendritic cells. Density plots indicate CD80 and CD86 expression of monocytes and dendritic cells matured with various cocktails of immunostimulatory compounds, leading to well-described types of maturation. **b** Expression of ID1, and more established markers used for characterization of monocytic MDSC, during myeloid cell maturation to a fully immunogenic phenotype associated with the indicated models for differentiation of human monocytes to mature dendritic cells

After demonstrating that increased ID1 expression in monocytic cells coincides with expression of phenotypic and immunosuppressive markers of monocytic MDSC, we wondered whether the opposite occurs when monocytic cells acquire an immunogenic phenotype. Therefore, we studied ID1 expression during myeloid cell maturation to a fully immunogenic phenotype, using various models for maturation of human monocyte-derived iDC to maDC. To this end, monocytes were isolated from healthy donor PBMC and treated with three different DC maturation cocktails (COMBIG [28], gold standard [26], and α-type 1 polarizing cocktail [27]). Proper maturation of monocyte-derived DC was confirmed by an increase in the percentages of CD80^+^CD86^+^ after exposure of iDC to maturation stimuli (Fig. 4a). During DC maturation, ID1 expression in CD11b^+^CD14^+^ cells significantly decreased, along with reduction in S100A8/9 and S100A9 expression (Fig. 4b). However, we did not see an increase in IRF8 expression after differentiation of monocytes to iDC or further maturation by exposure to any of the cocktails. Unexpectedly, DC matured with the gold standard cocktail actually expressed significantly lower IRF8 levels as compared to cultured monocytes (Fig. 4b).

## Discussion

Our analyzed patient cohort confirmed the immunosuppressive phenotype in CD33^+^CD11b^+^CD14^+^HLA-DR^low^  monocytic MDSC. Two immunosuppressive markers measured in our panel, iNOS and IDO, were expressed at significantly higher levels in HLA-DR^low^ monocytic MDSC, compared to HLA-DR^high^ monocytes. iNOS production is one of the pathways used by MDSC to inhibit T-cell activation [31, 32]. Our study shows that CD33^+^CD11b^+^CD14^+^HLA-DR^low^ monocytic MDSC have high expression of ID1 compared to CD33^+^CD11b^+^CD14^+^HLA-DR^high^ monocytes. Within the CD33^+^ CD11b^+^CD14^+^HLA-DR^low^ population, there was a strong correlation between ID1 and iNOS expression. This might indicate that ID1 is a regulator of iNOS production and may be associated with induction of molecular pathways for effector function of MDSC. Furthermore, iNOS expression has been shown to be an independent prognostic factor for overall survival (OS) in stage III malignant melanoma [33, 34]. However, we did not observe this in our study population, perhaps due to a relatively small sample size of patients with stage III melanoma. In other types of cancer than melanoma, ID1 showed promise as a prognostic marker for OS [11–13]. An explanation for the fact that we could not replicate these earlier findings in our patient group could be due to the relatively small size of the studied population.

No direct correlation between ID1 and iNOS has been described in the literature. However, it is thought that regulation of ID1 occurs through either the STAT5 or mTOR/Smad pathway [14, 35–37]. mTOR downregulation induces Smad and consequently also ID1 expression. mTOR downregulation has also been established as a stimulator for iNOS production [38]. With regards to our data, the hypothesis could then be formed that iNOS production by MDSC is regulated through the mTOR/Smad/ID1 pathway.

We show that both S100A8/9 and S100A9 have a strong positive correlation with ID1 expression, suggesting that they are, indeed, downstream effectors of ID1, as reported earlier [10]. The correlation of ID1 with S100A9 is not significant, in contrast to the correlation with S100A8/9. One reason can be that S100A9 monomers are highly unstable and, therefore, tend to form homodimers or heterodimers with S100A8 to increase stability [39]. Inhibition of downstream regulator S100A8/9 may help to promote tumor eradication as increased expression has been associated with tumor growth and MDSC accumulation [19, 20]. Both the correlation with immunosuppressive markers and downstream effectors involved in MDSC accumulation further strengthens our hypothesis that ID1 is part of the cascade that governs  accumulation and activatoin of monocytic MDSC. Therefore, ID1 could be a valuable biomarker for human monocytic MDSC in addition to the markers described in the literature [8]. However, the fact that no correlation was found between ID1 and either HLA-DR expression in CD33^+^CD11b^+^CD14^+ ^ cells or percentages of MDSC is not in line with the idea that ID1 is centrally involved in accumulation of MDSC.

In the B16F10 melanoma mouse model, IRF8 was shown to be downregulated in bone-marrow-derived myeloid cells in an ID1-dependent fashion by tumor-derived soluble factors, most notably TGF-β [10]. IRF8 is a transcription factor associated with driving myeloid differentiation towards a DC- and macrophage-like phenotype [40, 41]. Silencing of IRF8 in MDSC in a mouse model showed IRF8-mediated downregulation of FAS, thereby making MDSC less prone to elimination by T cells [42]. Thus, IRF8 not only skews myeloid cells towards a more DC-like and away from an MDSC-like phenotype, but is also able to increase their sensitivity to apoptosis. We showed that IRF8 is decreased in HLA-DR^low^ monocytic MDSC compared to HLA-DR^high^ monocytes in the blood of stage III and IV melanoma patients. However, we did not find a significant correlation between IRF8 and ID1 in our patient cohort, nor did we find a correlation between IRF8 and percentages of MDSC, which suggests that MDSC accumulation is not a direct consequence of IRF8 downregulation in myeloid cells. Furthermore, we did not see an increase in IRF8 expression after DC maturation with any of the cocktails used. Instead, DC matured with the gold standard cocktail actually expressed significantly lower IRF8 levels as compared to monocytes. This suggests that IRF8 upregulation is not associated per se with human DC differentiation, at least in our model based on the use of primary monocytes as starting material. Moreover, IRF8 downregulation does not seem to be ID1-dependent.

We showed that DC differentiation and maturation of monocytes derived from healthy donors go along with a decrease in ID1, S100A8/9, and S100A9. This indicates that ID1 downregulation is associated with differentiation of cells from the monocytic lineage to a more immunogenic phenotype, supporting the notion of ID1 as an important transcription factor in this process. However, from these experiments, it is still not possible to conclude whether ID1 is a key regulator of DC differentiation and maturation or merely a consequence of this. Knockdown and overexpression of ID1 in human myeloid cell cultures, either in primary cells or myeloid cell lines, are necessary to further establish ID1 function in this regard.

Evidence from literature indicates that ID1 expression can promote enhanced granulopoiesis [43]. This raises the question whether the increased ID1 expression seen in our study may point towards elevated production of granulocytic MDSC. Unfortunately, the samples in our study did not enable us to investigate this, as the freezing procedure that patient samples are subjected to negatively affect the granulocytic compartment. Therefore, this question could be addressed in a better way in follow-up studies on fresh patient samples.

In summary, our results indicate that ID1 might be a possible therapeutic target to deactivate monocytic MDSC and direct myeloid differentiation towards a less immunosuppressive and more immunogenic phenotype. Additional research has to be conducted to unravel the role of ID1 in the differentiation of monocytic cells towards either a DC or MDSC phenotype. Further studies by ID1 knockdown or by ID1 overexpression may shed more light on the functional role of ID1 in monocytes during myeloid differentiation. Together, these approaches may help to further establish the role of ID1 in myeloid cell differentiation and MDSC activation.

### Electronic supplementary material

Below is the link to the electronic supplementary material.

Supplementary file1 (PDF 399 kb)

## Abbreviations

DC: Dendritic cells
FBS: Fetal bovine serum
GM-CSF: Granulocyte–macrophage colony-stimulating factor
HLH: Helix–loop–helix
ID1: Inhibitor of differentiation 1
iDC: Immature dendritic cells
IDO: Indoleamine 2,3-dioxygenase
IFN-α: Interferon-α
IFN-γ: Interferon-γ
IL-1β: Interleukin-1β
IL-4: Interleukin-4
IL-6: Interleukin-6
iNOS: Inducible nitric oxide synthetase
IRF8: Interferon regulatory factor 8
MACS: Magnetic-activated cell sorting
maDC: Mature dendritic cells
MDSC: Myeloid-derived suppressor cells
PBMC: Peripheral blood mononuclear cells
PBS: Phosphate-buffered saline
PD-L1: Programmed death-ligand 1
PGE2: Prostaglandin E2
Poly I:C: Polyinosinic:polycytidylic acid
TGF-β: Transforming growth factor-β
TNF-α: Tumor necrosis factor-α
