# Supplementary material for: High expression of ID1 in monocytes is strongly associated with phenotypic and functional MDSC markers in advanced melanoma
Source: Cancer Immunol Immunother. 2020 Jan 17;69(4):513–22. doi: 10.1007/s00262-019-02476-9 (PMC7113206; doi:10.1007/s00262-019-02476-9)
Supplement: Supplementary file 1 — Supplementary file1 (PDF 399 kb) [file 262_2019_2476_MOESM1_ESM.pdf]

Supplementary figure 1

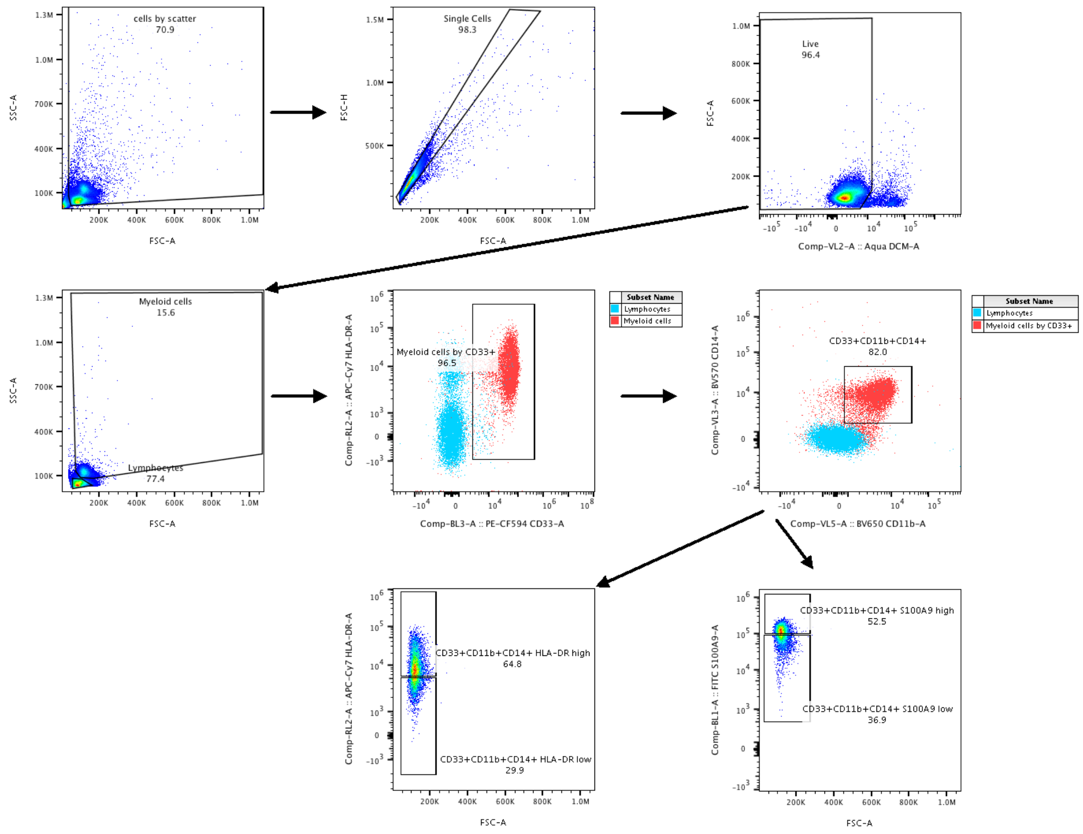

Supplementary fig. 1 Gating strategy for defining monocytic myeloid-derived suppressor cells, according to gating by forward and side scatter, CD33, CD11b, CD14 positivity and low tot absent HLA-DR expression. Low to negative expression of HLA-DR or S100A9 on $CD33^{+}CD11b^{+}CD14^{+}$ monocytes was defined using the lymphocyte population as an internal control, as the bulk of these cells are negative for HLA-DR. A subpopulation of activated T cells may express HLA-DR at relatively low levels, which was also seen in our samples.

Supplementary figure 2

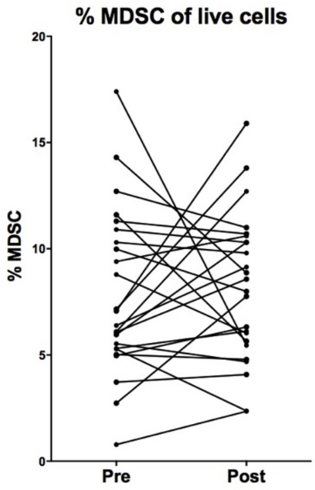

Supplementary fig. 2 Percentages of monocytic MDSC, defined as $CD33^{+}CD11b^{+}CD14^{+}HLA\text{-}DR^{low}$, of the live cell population in the blood of melanoma patients before and after surgical removal of melanoma metastases.

Supplementary figure 3

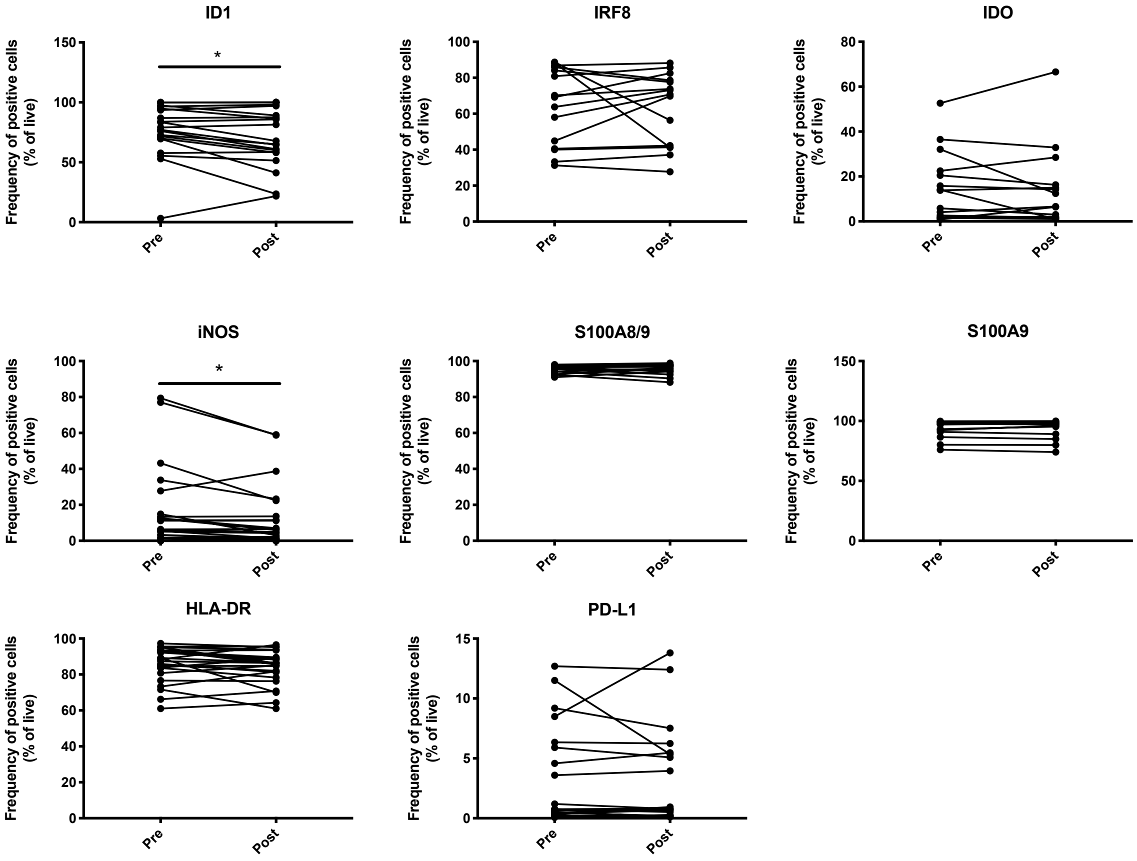

Supplementary fig. 3 Percentages of monocytes positive for the indicated markers in the blood of melanoma patients before and after surgical removal of melanoma metastases.

Supplementary figure 4

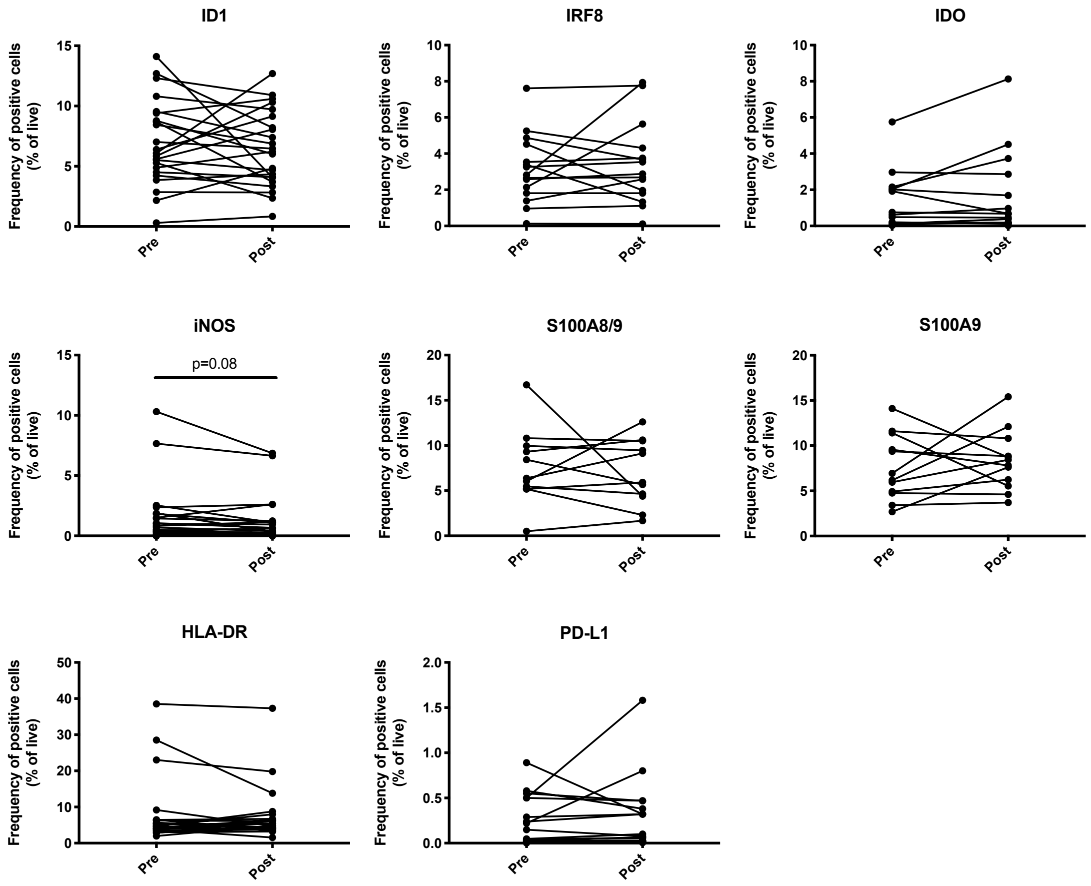

Supplementary fig. 4 Percentages of monocytic MDSC positive for the indicated markers within the live cell population in the blood of melanoma patients before and after surgical removal of melanoma metastases.
